# ITap: Index Finger Tap Interaction by Gaze and Tabletop Integration

**DOI:** 10.3390/s25092833

**Published:** 2025-04-30

**Authors:** Jeonghyeon Kim, Jemin Lee, Jung-Hoon Ahn, Youngwon Kim

**Affiliations:** 1 School of Computer Engineering, Kumoh National Institute of Technology, 61 Daehak-ro, Gumi-si 39177, Republic of Korea; dnrgusrla1@kumoh.ac.kr (J.K.); char3941@kumoh.ac.kr (J.L.); 2 Korea Electronics Technology Institute (KETI), 226 Cheomdan-gwagiro, Buk-gu, Gwangju 61005, Republic of Korea

**Keywords:** gaze-based interaction, hand tracking, virtual touchpad, ITap, tabletop interaction, object selection, scrolling, swiping gestures, multimodal interaction

## Abstract

This paper presents ITap, a novel interaction method utilizing hand tracking to create a virtual touchpad on a tabletop. ITap facilitates touch interactions such as tapping, dragging, and swiping using the index finger. The technique combines gaze-based object selection with touch gestures, while a pinch gesture performed with the opposite hand activates a manual mode, enabling precise cursor control independently of gaze direction. The primary purpose of this research is to enhance interaction efficiency, reduce user fatigue, and improve accuracy in gaze-based object selection tasks, particularly in complex and cluttered XR environments. Specifically, we addressed two research questions: (1) How does ITap’s manual mode compare with the traditional gaze + pinch method regarding speed and accuracy in object selection tasks across varying distances and densities? (2) Does ITap provide improved user comfort, naturalness, and reduced fatigue compared to the traditional method during prolonged scrolling and swiping tasks? To evaluate these questions, two studies were conducted. The first study compared ITap’s manual mode with the traditional gaze + pinch method for object selection tasks across various distances and in cluttered environments. The second study examined both methods for scrolling and swiping tasks, focusing on user comfort, naturalness, and fatigue. The findings revealed that ITap outperformed gaze + pinch in terms of object selection speed and error reduction, particularly in scenarios involving distant or densely arranged objects. Additionally, ITap demonstrated superior performance in scrolling and swiping tasks, with participants reporting greater comfort and reduced fatigue. The integration of gaze-based input and touch gestures provided by ITap offers a more efficient and user-friendly interaction method compared to the traditional gaze + pinch technique. Its ability to reduce fatigue and improve accuracy makes it especially suitable for tasks involving complex environments or extended usage in XR settings.

## 1. Introduction

Gaze input is an intuitive interaction method that enables users to point to objects with minimal physical effort. It has been extensively utilized in both academic research and commercial applications. Recent research increasingly explores multimodal interactions that integrate gaze with other input modalities to enhance the overall user experience [1,2,3,4]. A notable example of this multimodal approach is the gaze + pinch interaction employed in devices such as Apple Vision Pro, where users direct their gaze toward an object and select or manipulate it through a pinch gesture.

However, the gaze + pinch interaction paradigm has several limitations. First and foremost is the issue of gaze inaccuracy. Despite significant advances in gaze tracking accuracy, users often encounter difficulties accurately targeting objects under certain conditions [5,6]. Specifically, misalignment of the user’s eyes or improper calibration can cause substantial inaccuracies in gaze-based interaction [7]. Additionally, selecting small, distant, or densely clustered objects requires considerable effort [8,9,10,11].

The pinch gesture itself faces several challenges as well. Unintended activation: Since the pinch gesture is detected when users bring their fingers together, natural hand movements can be mistakenly recognized as pinch gestures, leading to unintended activations. Such misrecognition undermines interaction accuracy and degrades the overall user experience [12,13]. Lack of haptic feedback: Although the pinch gesture involves grasping, it lacks tactile confirmation essential for ensuring correct selection or activation. This limitation is particularly evident for interactions such as swiping or scrolling, where the absence of feedback can cause uncertainty regarding whether inputs have been registered or gestures executed correctly [14,15]. Wrist fatigue: Prolonged performance of the pinch gesture can result in wrist strain, particularly during continuous scrolling or swiping actions. Deviating from the wrist’s natural resting position during mid-air gestures contributes to wrist fatigue, often referred to as “gorilla arm syndrome” [16,17]. Extended use of these gestures, characterized by raising arms for prolonged periods, can lead to discomfort and potential long-term strain [18].

**Figure 1:**
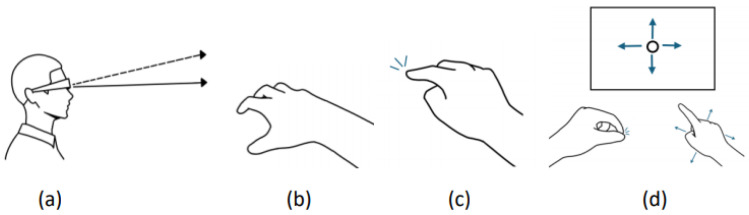
ITap is a tabletop interaction that utilizes hand-tracking technology to facilitate interaction on the tabletop. The following actions are involved: (**a**) pointing with gaze, (**b**) the hand gesture that generates a virtual touchpad on the table surface, (**c**) touching the table surface, and (**d**) the use of manual mode, wherein a pinch gesture with the opposite hand halts the gaze ray, allowing the user to manipulate the cursor by moving their right hand in different directions (indicated by arrows).

To address these limitations, we propose a novel interaction technique, ITap. ITap leverages hand-tracking technology, allowing users to create a virtual touchpad on a tabletop surface and perform interactions by tapping with their index finger (Figure 1). In this study, we conducted two experiments to evaluate the performance and usability of ITap. The first experiment compared ITap’s manual cursor mode with the traditional gaze + pinch method, focusing on object selection time and error rates at various distances. The second experiment assessed user comfort and fatigue during scrolling and swiping tasks and collected subjective feedback regarding the user experience of both interaction methods. In light of these considerations, the primary goal of this research is to propose and validate an interaction technique that addresses the limitations of current gaze-based input methods. Specifically, we aim to answer the following research questions:

(1) Does ITap’s manual cursor mode achieve superior target selection performance, in terms of speed and accuracy, compared to the traditional gaze + pinch interaction method?
(2) Does ITap offer improved usability, comfort, and reduced cognitive load in scrolling and swiping tasks compared to conventional pinch-based gestures?

By systematically investigating these questions, our study aims to contribute valuable insights into designing more effective, precise, and ergonomically optimized interaction techniques for extended reality (XR) environments.

## 2. Related Work

### 2.1. Touch-Based Interaction

Touch interaction is a pervasive and fundamental input method widely adopted in modern devices, with growing exploration within virtual reality (VR) environments. Research on touch-based interaction techniques aims to extend the capabilities of touch input within augmented reality (AR) by creating virtual touchpads on various surfaces [4,19]. One notable innovation is TriPad, a system enabling users to perform touch input on ordinary surfaces via hand tracking. TriPad facilitates interactions by allowing users to perform finger movements in mid-air, thus providing a versatile input mechanism tailored for immersive environments [4]. Additionally, various sensor-based touch interaction technologies have been investigated extensively [20,21,22,23,24]. For instance, TapID employs wearable sensors to facilitate swift and intuitive touch interactions in VR, detecting finger movements in real time and translating them into inputs for virtual environments. Similarly, FingerTouch uses sensors attached to the user’s fingernails to detect touch inputs within AR environments. By accurately recognizing finger taps and gestures, these sensors convert tactile interactions into precise inputs for interacting with AR displays [23].

TriPad and ITap both utilize physical surfaces for interaction, yet differ significantly in their respective approaches and intended application environments. TriPad primarily leverages hand-tracking technology, enabling touch input on conventional surfaces by recognizing users’ mid-air finger movements. This approach provides versatile input capabilities without necessitating direct physical contact, enhancing its applicability in immersive scenarios. In contrast, ITap combines physical table-based touch input with gaze-based selection, specifically tailored for extended reality (XR) environments. ITap enables users to perform interactions by physically tapping a tabletop surface while simultaneously employing gaze direction to select targets, thus enhancing both precision and intuitiveness. This integration constitutes a novel interaction approach distinct from TriPad’s reliance solely on hand tracking. By merging gaze and touch inputs, ITap significantly enhances the speed and accuracy of target selection.

Existing touch-related research has primarily focused on mapping physical surfaces to digital screens at a 1:1 ratio, whereby selecting a coordinate on the physical surface directly corresponds to the same coordinate on a display, or overlaying digital interfaces onto tabletop surfaces. Moreover, previous gaze-based interaction studies have lacked effective techniques to compensate for gaze-tracking inaccuracies, with trigger input methods typically relying on 3D inputs that require users to operate hands or controllers mid-air. Such approaches lead to user fatigue, especially during extended usage periods. In contrast, ITap extends traditional touch interaction to tabletop environments, allowing users to select and manipulate virtual objects by tapping a physical surface with their index finger (analogous to a mouse click). This method offers an intuitive interface reminiscent of mouse-based interactions and significantly reduces user fatigue by converting typical 3D mid-air gestures into simpler 2D surface interactions. Furthermore, ITap includes a dedicated manual mode to address the inherent inaccuracies associated with gaze-based interaction, providing a more precise and reliable input method.

### 2.2. Gaze-Based Interaction

Gaze-based multimodal interaction represents a nascent and promising research domain within virtual reality (VR). This emerging technology leverages gaze input, enabling users to seamlessly control cursor navigation and object selection merely by directing their gaze, thereby reducing reliance on additional input devices and physical exertion [11,25]. Such gaze-based methods offer an intuitive and efficient user experience, which is particularly beneficial in immersive VR environments. A commercially available example of this technology is the gaze + pinch method, wherein users initially direct their gaze toward a target object and subsequently perform a two-finger pinch gesture to initiate interactions. Once visual engagement with the target object is established, the pinch gesture facilitates further manipulation, resulting in a seamless and comfortable interaction experience [1].

The gaze + pinch method derives from the gaze–touch concept, integrating gaze-based pointing with touch gestures on the same interaction surface to facilitate intuitive and fluid interactions [26]. A related approach was proposed in [26], where users select objects by directing their gaze onto a 2D surface and confirming actions through touch gestures, thus introducing an innovative combination of gaze and touch inputs.

Building upon this concept, CursorShift combines gaze pointing with direct touch input, providing users with precise control and flexibility within 2D tablet environments. This technique enables users to specify approximate locations using touch input and subsequently refine their interactions with gaze, significantly enhancing interaction efficiency in 2D contexts [4]. Additionally, the Manual and Gaze Input Cascaded (MAGIC) pointing system explored integrating gaze-based coarse targeting with manual fine-tuning input [27]. This pioneering exploration into combined gaze and manual inputs laid the groundwork for developing more sophisticated multimodal interaction methods.

Another notable technique is Gaze–Hand Alignment, which combines gaze tracking with mid-air hand pointing to enable precise and intuitive interactions within augmented reality (AR) environments [28]. Users employ their gaze to direct attention toward objects or interface elements, while simultaneously using hand movements as a secondary input to refine or confirm selections. This combination facilitates seamless and efficient interaction by reducing the cognitive and physical effort required for accurate targeting. Unlike traditional gaze-only selection techniques, which can suffer from inaccuracies due to eye-tracking limitations, Gaze–Hand Alignment leverages complementary hand inputs for enhanced precision, thereby improving the overall user experience. This approach is especially beneficial for tasks requiring fine-grained control, such as menu navigation, object manipulation, and virtual workspace interactions, effectively mitigating errors associated with gaze-only methods [2].

Recent advancements further expand gaze-based interactions. Pfeuffer et al. detailed principles and challenges of gaze and pinch interaction specifically within XR environments, highlighting critical design factors for multimodal usability [21]. Wang et al. proposed EyeSQUAD, utilizing gaze-based progressive refinement for accurate 3D selection tasks, overcoming common gaze inaccuracies through sequential refinement techniques [29]. Additionally, McDonald et al. presented SmartVR Pointer, an innovative integration of smartphone inputs and gaze orientation for intuitive VR navigation and selection tasks [30]. Tian et al. demonstrated how gaze can efficiently complement touch input in AR scenarios, effectively balancing speed and accuracy through hybrid gaze–touch interactions [31]. Jeong et al. introduced GazeHand, facilitating interaction with distant objects through gaze-based targeting coupled with virtual hand manipulation [32]. Chen et al. investigated predicting user interaction intent through gaze and gesture data, enhancing the responsiveness and fluidity of gaze-driven interfaces [33].

## 3. ITap Methodology

### 3.1. ITap

**Figure 2:**
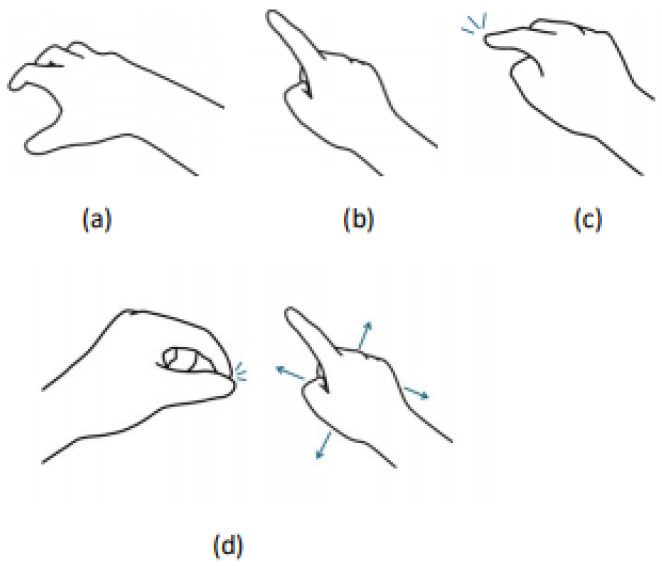
ITap’s Hand Gesture (**a**) refers to the hand gesture used to create a virtual touchpad. A touchpad is generated by holding the hand briefly and then tapping the table surface. (**b**) shows a scenario where the touchpad has been generated but no touch has been made. (**c**) demonstrates the act of touching the table surface. (**d**) shows the use of the manual mode, where a pinch gesture with the opposite hand stops the beam, allowing the user to move the cursor by moving the right hand in various directions, as indicated by the arrows.

ITap is a technology that creates a virtual touchpad on a tabletop surface, enabling user interaction through finger-based touch input. Figure 2 illustrates the overall workflow and operation of ITap. Initially, users place their hand briefly on the tabletop surface, creating a virtual touchpad and setting the interaction to a ready state (Figure 2a). Once the virtual touchpad is activated, the user’s hand remains positioned above the tabletop, representing a state where the touchpad is active but no touch input has yet occurred (Figure 2b). When the user subsequently touches the tabletop surface with their finger, the system registers this as input and supports various gestures beyond simple tapping, including dragging and swiping (Figure 2c).

Additionally, users can precisely control the cursor by performing a pinch gesture using the opposite hand to activate the manual mode. This manual mode enables users to accurately reposition the cursor by freely moving their hand across the surface, and it is deactivated once the pinch gesture is released (Figure 2d). This sequential interaction demonstrates ITap’s key elements, including touchpad activation, finger-based touch input, gaze-based selection, and precise manual control.

Specifically designed for tabletop interactions, ITap integrates gaze-based input with touch interactions, allowing users to select objects with their gaze and manipulate them by tapping the virtual pad. ITap builds upon core principles of gaze–touch and extends them into XR environments. By combining gaze and touch inputs, ITap significantly improves interaction intuitiveness and efficiency. The gaze–touch paradigm, originally developed for 2D surfaces, enables users to point at objects using their gaze and confirm actions via touch gestures, considerably enhancing the user experience [26]. Leveraging these principles, ITap introduces a virtual touchpad on physical tabletops, enabling precise and natural interactions within XR scenarios.

Unlike traditional gaze + pinch techniques, which require users to lift their arms into mid-air when performing actions such as scrolling or swiping, ITap enables users to comfortably rest their arms on the tabletop throughout these interactions. Although conventional gaze + pinch interactions can initially be performed with arms resting on surfaces, mid-air actions like scrolling and swiping still necessitate lifting the arm, leading to discomfort during prolonged use. ITap addresses this limitation by facilitating scrolling and swiping gestures while maintaining arm support on the tabletop.

Furthermore, the choice to use the opposite-hand pinch gesture in ITap is intentional. Although a conventional pinch gesture can be performed with the arm resting on a tabletop, it nonetheless requires mid-air movements for scrolling and swiping actions. Conversely, the opposite-hand pinch gesture eliminates the need for mid-air arm movements, providing a more stable and comfortable interaction experience. By effectively integrating tabletop inputs with mid-air gestures, ITap enhances usability and addresses fatigue issues typically associated with prolonged mid-air interactions [21]. This approach is particularly advantageous for 2D interface tasks within VR environments, such as web browsing or data navigation, as it closely mirrors the familiar user experience of interacting with a tablet or monitor [27,34]. Additionally, the tactile feedback provided by the tabletop surface stabilizes gestures like scrolling, swiping, and dragging, reducing the risk of developing “gorilla arm syndrome”, a common issue resulting from extended mid-air interactions [26].

### 3.2. ITap Design Consideration

ITap utilizes hand-tracking technology to detect touch interactions directly on a tabletop surface, eliminating the need for external sensors or additional input devices. Despite significant advances in hand-tracking capabilities, several challenges persist regarding tracking accuracy [35]. Initially, multi-finger interaction methods were explored; however, from the perspective of head-mounted displays (HMDs), fingers such as the middle, ring, and little fingers are frequently occluded, resulting in decreased tracking reliability. Consequently, the thumb and index finger emerged as primary input choices, with the index finger identified as the most natural option for touch interactions [23].

The manual mode in ITap introduces a novel approach to seamlessly transition between gaze-based pointing and fine cursor adjustments using a pinch gesture with the left hand. This gesture was intentionally selected for its intuitive nature, closely mimicking the action of physically grasping an object, thus enhancing user understanding and ease of adoption [20]. The pinch gesture temporarily holds the gaze cursor, enabling smooth transitions between gaze-driven pointing and manual adjustments without interrupting the user’s workflow. When the manual mode is activated through the left-hand pinch gesture, the user can precisely adjust cursor positioning by moving their right hand across the tabletop surface. Specifically, movements along the x- and z-axes of the right hand translate directly into cursor movements along the x- and y-axes, effectively replicating the intuitive operation of a traditional computer mouse. Leveraging this familiar mouse-based interaction paradigm ensures that ITap delivers a highly intuitive and accessible user experience, significantly reducing the learning curve for new users.

This design builds upon previous research exploring gaze- and touch-based interactions. For example, the “Look And Touch” interaction approach demonstrated that combining gaze-based pointing with touch gestures significantly improves interaction accuracy and provides tactile confirmation, particularly within 2D environments [34]. Extending these findings, ITap employs the left-hand pinch gesture as a natural and ergonomic method for momentarily pausing the gaze cursor, facilitating precise cursor adjustments without disrupting ongoing tasks. By assigning the pinch gesture to the non-dominant hand (typically the left hand), the dominant hand remains free for primary interactions, such as tapping or object manipulation, thus offering ergonomic and practical benefits.

Furthermore, the practical advantages of the pinch gesture specifically reflect the characteristics inherent to tabletop interaction environments. When hands rest naturally on a table surface, thumb and index finger tracking from the HMD perspective is more reliable, whereas middle, ring, and little fingers often become occluded or fall outside the effective tracking field.

### 3.3. Implementation

ITap uses hand-tracking technology to detect touch interactions directly on a tabletop surface without relying on external sensors or additional devices. The system was developed using Unity and C#, and evaluated using the Meta Quest Pro platform. It integrates Meta’s hand-tracking API along with built-in gaze-tracking functionality.

**Figure 3:**
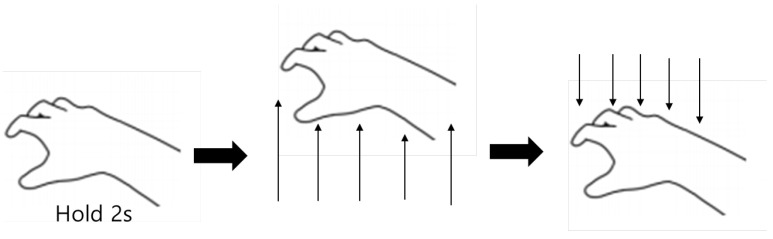
How to make a virtual touchpad.

The operational workflow of ITap is as follows (Figure 3): The interaction begins when the user places their hand flat on the tabletop surface and holds it stationary for two seconds, prompting the system to register a ready state for creating a touchpad. Subsequently, the user taps the surface again to activate the virtual touchpad. This standby state is activated exclusively when all five fingers are simultaneously in contact with the tabletop, with each finger joint aligned. Any deviation from this precise hand position results in the deactivation of the standby state. To prevent accidental activation of the touchpad, a distinct tap gesture was implemented to explicitly indicate the user’s intention. Since the y-coordinate of the virtual touchpad is predefined, ITap registers touch inputs whenever the user’s index finger reaches this specified height. The input is disengaged as soon as the fingertip is lifted from the surface. Due to inherent inaccuracies associated with hand tracking [35], the y-coordinate can vary slightly even for identical gestures. Therefore, a suitable correction factor for the touchpad’s y-coordinate was empirically determined through internal testing to ensure reliable and consistent performance.

The gaze + pinch interaction technique was implemented by integrating gaze tracking and pinch gestures to facilitate precise target selection. This implementation utilized the Meta Quest Pro’s built-in gaze tracking and hand-tracking APIs, thereby ensuring high accuracy in detecting user interactions. For gaze tracking, the Meta Quest Pro’s gaze-tracking API was employed to collect gaze data and detect target fixation. To distinguish intentional fixation from casual gaze movements, users were required to maintain gaze fixation on the target for at least 0.2 s for it to become selectable. Notably, prior work such as GazeRayCursor [36] employed a 0.3-s dwell time for similar VR target selection tasks. In contrast, our study adopted a shorter threshold of 0.2 s to enable faster interaction while still maintaining sufficient reliability in detecting user intent.

For detecting pinch gestures, the Meta Quest Pro’s hand-tracking API was utilized to precisely track finger movements. A pinch gesture was recognized when the distance between the thumb and index finger was reduced to less than 0.1 cm and subsequently released. This threshold was carefully chosen to accurately detect natural pinch gestures while filtering out unintended or casual hand movements.

For gaze input, the system averaged gaze vectors from both eyes, with the resulting gaze ray originating from the midpoint between the eyes. Specifically, the gaze-tracking implementation utilized Meta Quest Pro’s built-in eye-tracking API, which provides real-time rotational data from each eye. Leveraging this functionality, the system computed the gaze direction by integrating the rotational data from both eyes to produce a precise gaze ray corresponding to the user’s line of sight. Additionally, prior to conducting experiments, each participant’s gaze was calibrated using the Meta Quest’s built-in gaze calibration function. During calibration, participants fixated sequentially on a series of predefined points displayed within the virtual environment. This procedure aligned gaze tracking data to each individual’s unique eye anatomy, effectively accounting for personal differences in eye configurations.

The interaction sequence commenced with users fixating their gaze on the intended target for 0.2 s, thereby signaling their intent to select it. While maintaining gaze fixation, users performed a pinch gesture by reducing the distance between thumb and index finger to less than 0.1 cm. For successful selection, this pinch gesture needed to occur within 500 ms following gaze fixation, ensuring intentional and efficient selection.

The chosen parameters were meticulously determined, guided by considerations from usability principles and insights drawn from prior research. The 0.2-s gaze fixation duration was established as an optimal balance to minimize accidental selections while maintaining task efficiency. Likewise, the 0.1 cm pinch-distance threshold ensured natural, precise gesture recognition, taking advantage of the Meta Quest Pro’s highly accurate hand-tracking capabilities. Collectively, these parameters enabled a seamless integration of gaze and pinch inputs, facilitating reliable and efficient target selection.

## 4. Experiment

### 4.1. Ethical Considerations

This study was conducted in accordance with the ethical standards of the Kumoh National Institute of Technology and was approved by the Bioethics Review Committee. The details of the approval are as follows:

- Ethics Committee Name: Bioethics Review Committee, Kumoh National Institute of Technology
- Approval Code: 202412-HR-011-01
- Approval Date: 26 December 2024

All participants involved in the study were fully informed about the research purpose, procedures, potential risks, and their rights as participants. Written informed consent was obtained from all subjects before participation in the study.

### 4.2. Participants and Experimental Setup

A total of 15 participants took part in this study, including 11 males and four females, aged between 20 and 34 years. The participants’ VR experience levels were categorized as follows: four advanced VR users, three intermediate users, and eight beginners. Experiments were conducted using the Meta Quest Pro, a device equipped with built-in eye-tracking capabilities. Participants were seated in front of a physical table, and a virtual touchpad was initialized before commencing the experiments.

### 4.3. Experiment 1: Target Selection Accuracy and Speed

**Figure 4:**
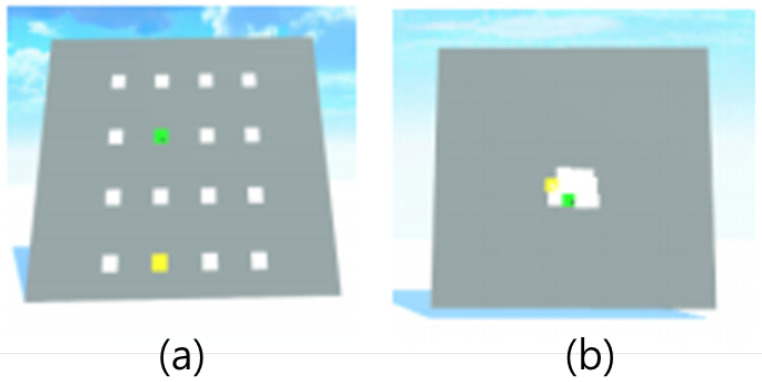
Experimental setups for Experiment 1: (**a**) target selection at varied distances, where colored boxes represent target objects placed at near, medium, and far distances. (**b**) disambiguation task in a densely arranged layout, where color-coded boxes represent closely positioned or overlapping targets to simulate ambiguity. Across both settings, yellow boxes indicate the goal object, while green boxes highlight the object currently selected by the user.

The first experiment compared the traditional gaze + pinch technique with ITap’s manual mode, focusing on pointing accuracy and speed. Conducted in a virtual reality (VR) environment, participants visually tracked changes in target objects, objects under gaze, and selected objects. The experimental setup consisted of a virtual wall measuring 11 m × 9 m, with targets arranged in a structured 4 × 4 grid. Each target measured 0.5 m × 0.5 m and was placed 2.3 m away from each corner of the wall, maintaining 1 m spacing between adjacent targets (Figure 4a).

Participants performed selections from distances of 3 m, 6 m, 9 m, 12 m, 15 m, 18 m, 21 m, 24 m, 27 m, and 30 m. Each participant completed 160 tasks per interaction technique, resulting in a total of 320 tasks across both techniques. Task completion time (TCT) and error rate served as key performance metrics.

Additionally, participants performed a second task within this experiment to evaluate selection accuracy in densely arranged scenarios. Targets were randomly overlapped within a confined area of 2 m × 1.5 m from a fixed distance of 18 m, significantly increasing selection complexity (Figure 4b). Participants completed 16 distinct selection tasks, each repeated twice for both interaction techniques. Task completion time (TCT) and error rate were again primary metrics.

### 4.4. Experiment 2: Scrolling and Swiping Usability

The second experiment directly compared usability and subjective user experience between the pinch gesture and ITap’s tabletop gesture, focusing specifically on scrolling and swiping tasks.

**Figure 5:**
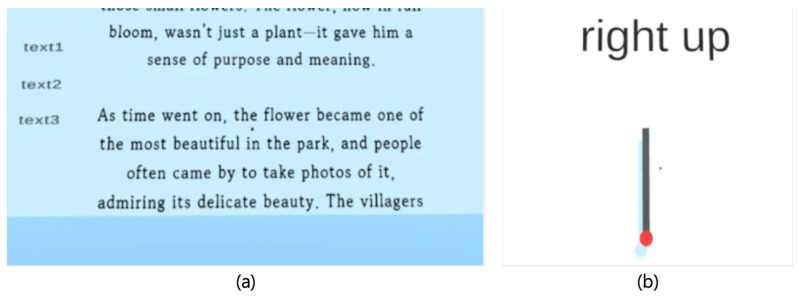
Experimental setups for Experiment 2: (**a**) scrolling task with textual content, and (**b**) swipe task directional indicators.

In the scrolling task, participants interacted with three types of textual content such as a short novel, an article, and an informational passage (Figure 5a). Participants performed approximately 60 scrolling gestures at intervals of 23 cm, with scrolling actions registered only if gaze remained within the text area. This design aimed to simulate realistic usage and assess perceived comfort, usability, and fatigue.

The swipe task required participants to perform gestures in eight different directions: up, upper-right diagonal, right, lower-right diagonal, down, lower-left diagonal, left, and upper-left diagonal (Figure 5b). Directions were randomized, and each participant completed a total of 32 swipes per interaction technique. Subjective feedback regarding comfort, naturalness, and fatigue was collected.

### 4.5. Limitations for Experiment

Although the current experimental design offers a holistic evaluation of ITap and gaze + pinch techniques within VR settings, several limitations should be acknowledged. First, the study did not employ Fitts’ law, a widely recognized model for evaluating pointing and selection tasks. The absence of this standardized framework limits the comparability of our findings to existing research on gaze-based and touch-based interactions. Consequently, directly comparing ITap’s performance with other techniques evaluated through Fitts’ law is challenging.

Second, the complexity inherent in the 3D selection tasks introduces additional cognitive and perceptual factors not accounted for by Fitts’ law. The varied target distances and spatial arrangements required participants to dynamically adjust their gaze and hand movements. As a result, measured task completion times and error rates may reflect factors beyond the fundamental efficiency of the interaction methods themselves, such as the cognitive load associated with navigating complex 3D environments.

Third, the selection tasks used in this study were designed to simulate practical scenarios, but they may not fully generalize to all XR applications. The experiments involved selecting targets arranged in a fixed 4 × 4 grid at predetermined distances, which may differ from target distributions, occlusions, or dynamic environmental conditions found in diverse real-world contexts. Thus, interaction performance observed in this study may not directly translate to other application scenarios.

Lastly, the lack of supplementary evaluation using Fitts’ law results in no direct benchmark for comparing ITap and gaze + pinch against traditional selection models. While this research emphasizes practical interactions within XR environments, integrating Fitts’ law-based metrics in future studies could provide a standardized basis for comparing selection efficiency across various interaction methods.

## 5. Result

### 5.1. Selection Facilitation Task Completion Time

**Figure 6:**
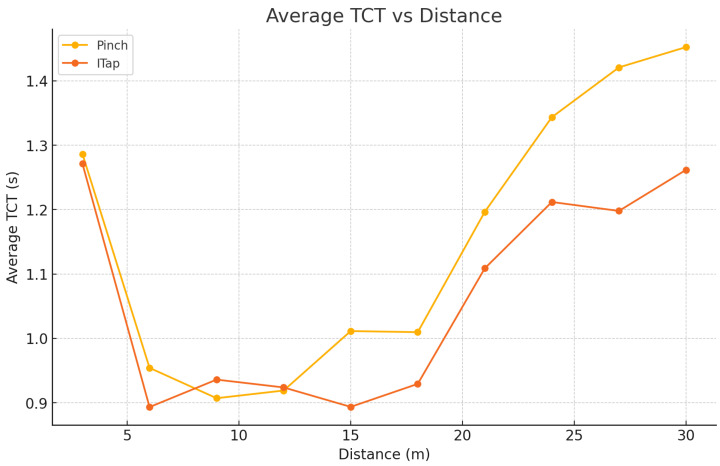
Graph of average TCT for ITap and Pinch by distance.

The results demonstrate that ITap consistently provided stable and faster selection times, maintaining significantly lower error rates than the gaze + pinch method, particularly at longer distances (see Figure 6). A detailed analysis by distance intervals clearly illustrates how performance differences evolved across the short-, medium-, and long-distance conditions.

**Figure 7:**
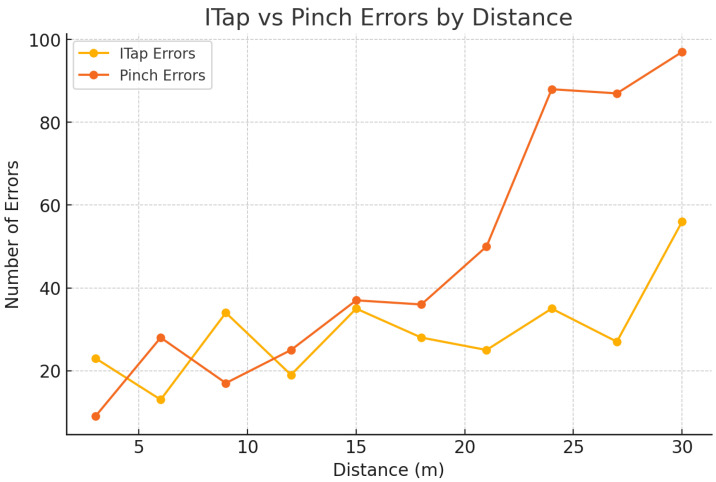
Graph of average errors for ITap and Pinch by distance.

At short distances (3 to 6 m), the performance differences between ITap and gaze + pinch were relatively minor, primarily due to the inherently higher gaze tracking accuracy achievable at close ranges. Specifically, selection times for the gaze + pinch method ranged from 1.37 to 1.49 s, accompanied by moderate error rates ranging from 2.5% to 3.2%. In comparison, ITap showed slightly faster and more consistent performance, with selection times between 1.12 and 1.15 s and error rates substantially lower, from 1.0% to 1.2%. Although ITap’s manual adjustment capability did not provide a significantly large advantage at these shorter distances, the reduced physical effort associated with the tabletop-based gesture interaction helped maintain consistently superior performance. Additionally, both methods exhibited slightly longer selection times and slightly increased error rates at the closest tested distance of 3 m, likely due to participants needing to make additional minor adjustments in gaze alignment at close ranges, highlighting inherent limitations of gaze-only selection methods (Figure 7).

At medium distances (9 to 18 m), performance discrepancies between ITap and gaze + pinch became significantly more pronounced. The gaze + pinch method’s performance notably deteriorated, with average selection times increasing sharply from 1.80 s up to 2.78 s as distance increased. Correspondingly, the error rates for gaze + pinch also demonstrated a marked rise, escalating from 4.8% to 12.3%. This performance decline can be primarily attributed to the reduced accuracy of gaze tracking at intermediate distances, necessitating frequent gaze adjustments and repeated corrective pinch gestures, thereby negatively impacting overall user efficiency.

In stark contrast, ITap demonstrated remarkable stability and reliability across these medium distances. Selection times remained consistently short, ranging from 1.18 to 1.35 s, while error rates remained consistently low, between 1.5% and 2.2%. The manual cursor repositioning mode of ITap effectively compensated for gaze tracking inaccuracies, enabling users to quickly and intuitively correct minor gaze deviations and confidently select the intended targets. Consequently, ITap provided a significantly improved user experience characterized by greater predictability and less frustration, highlighting clear ergonomic and performance benefits over gaze + pinch at intermediate distances.

At long distances (21 to 30 m), differences in performance became most pronounced, underscoring ITap’s substantial advantage in scenarios that demand precise target selection from afar. The gaze + pinch method exhibited a drastic reduction in usability and accuracy at these distances, with average selection times increasing sharply from 3.40 s up to 5.83 s. Additionally, error rates surged dramatically, ranging from 15.7% up to an extremely high 27.8%. This severe decline in performance is primarily due to significantly compromised gaze-tracking accuracy at longer distances, as minor head or eye movements resulted in substantial shifts in gaze points, leading to frequent mis-selections and multiple corrective attempts.

Statistical Validation

A two-way ANOVA confirmed the significant effects of the interaction method and distance on both selection times and error rates.

Selection Times:

(1) F(1,4780)=27.45, p<0.001

(2) F(9,4780)=49.49, p<0.001

Error Rates:

(3) F(1,4780)=34.12, p<0.001

(4) F(9,4780)=62.45, p<0.001

A significant interaction effect between method and distance was also observed for both metrics:

(5) F(9,4780)=2.40, p=0.01

(6) F(9,4780)=3.12, p=0.002

Tukey HSD post hoc analysis confirmed ITap’s superior performance across all distances, particularly at longer ranges.

### 5.2. Disambiguation Task Performance

**Figure 8:**
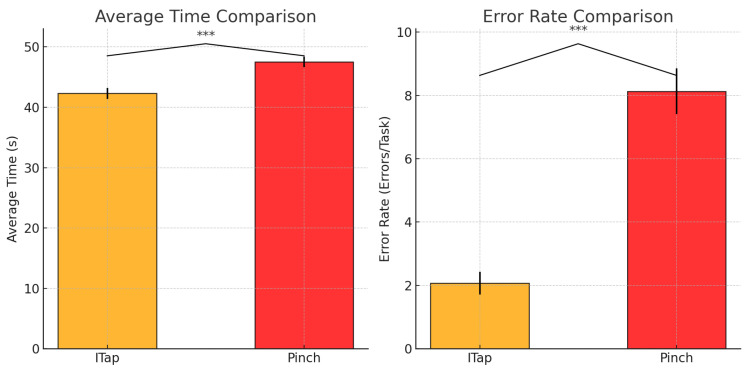
Graph of average selection time and error rates for Pinch and ITap methods in disambiguation tasks. Colors indicate interaction methods (red = Pinch, orange = ITap), and asterisks (***) denote statistically significant differences with *p* < 0.001.

The ITap method demonstrated significantly faster target selection times and lower error rates compared to the Pinch method (Figure 8). ITap reduced execution time by 11%, with an average of 42.31 s (SD = 3.40) compared to 47.52 s (SD = 3.36) for Pinch. An independent samples *t*-test confirmed this difference as statistically significant (*t* = −4.15, *p* < 0.001). Regarding error rates, ITap had an average error rate of 2.07 (SD = 1.33), significantly lower than the Pinch method’s average of 8.13 (SD = 2.80), as confirmed by an independent samples *t*-test (*t* = −7.53, *p* < 0.001).

These results highlight ITap’s effectiveness in enhancing user interaction precision and speed, particularly through its manual mode that effectively mitigates gaze inaccuracies, substantially improving selection accuracy and reducing errors compared to the Pinch method.

### 5.3. Usability Evaluation

**Figure 9:**
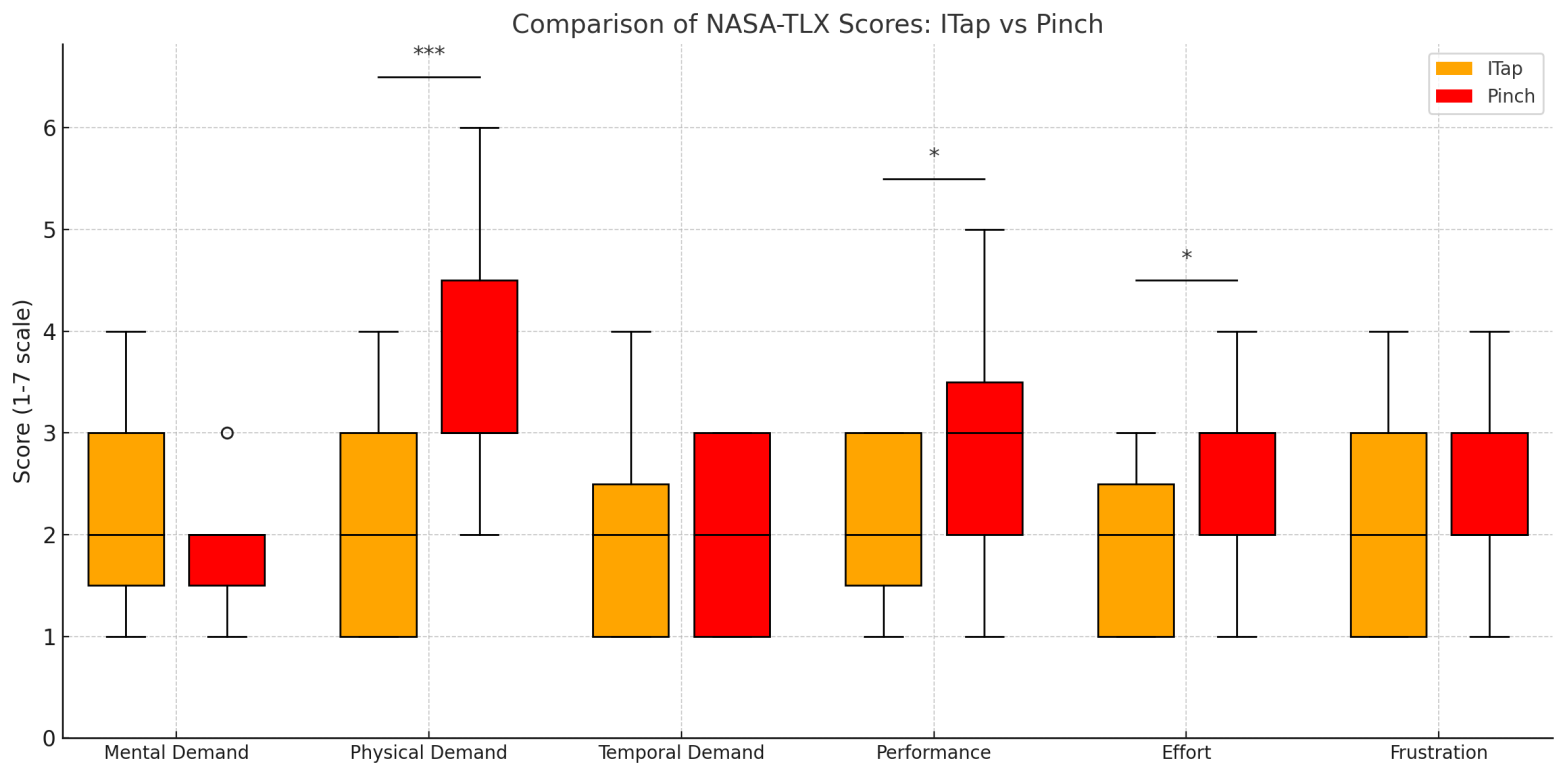
Graph of user evaluations for the NASA-TLX survey. Asterisks indicate statistically significant differences (*: *p* < 0.05, ***: *p* < 0.001), and circles represent outliers.

Following the completion of the scrolling and swipe tasks using both the ITap and Pinch interaction methods, we conducted independent *t*-tests to compare the results of the NASA-TLX evaluations across various dimensions (Figure 9). The analysis revealed statistically significant differences between the two methods in terms of Physical Demand, Performance, and Effort. Physical Demand: The Pinch method imposed a significantly higher physical burden on the participants than the ITap method (*t* = −3.83, *p* < 0.001). This suggests that the Pinch method requires greater physical exertion from users than the ITap method. Performance: ITap was rated significantly higher in terms of performance (*t* = −2.33, *p* = 0.027), indicating that ITap enables more effective task completion. Effort: The ITap method required significantly less effort than the Pinch method (*t* = −2.30, *p* = 0.029), indicating that ITap requires less energy from users to complete the same tasks.

## 6. Discussion

### 6.1. Experiment 1: Target Selection and Disambiguation

The results from Experiment 1 clearly demonstrate the ergonomic and usability advantages of the ITap method over the gaze + pinch method, particularly for selection tasks requiring precision across various distances. ITap consistently exhibited significantly faster selection times and lower error rates, especially at medium to long distances where gaze inaccuracies are more prevalent, aligning with findings from previous studies on gaze inaccuracies at extended ranges [5,6,7]. ITap’s manual cursor adjustment effectively compensated for gaze tracking inaccuracies, enabling precise and consistent selections even under challenging conditions, corroborating similar multimodal interaction studies that integrated manual adjustments for improved accuracy [27].

Additionally, the disambiguation task results further highlight ITap’s effectiveness. The significantly reduced execution time and lower error rates underscore ITap’s capability to mitigate inherent inaccuracies of gaze-based selection discussed in the prior literature [3,11,26]. By enabling precise target selection through manual adjustments, ITap substantially reduces the likelihood of errors and repeated selections, thus improving overall task efficiency and user satisfaction, echoing results from other gaze–touch multimodal interfaces [4,26]. These findings clearly address our primary research question, affirming that ITap’s manual mode outperforms traditional gaze-based gestures (gaze + pinch) regarding selection speed and accuracy, especially in scenarios involving complex or densely arranged targets.

### 6.2. Experiment 2: Usability in Scrolling and Swiping Tasks

The findings from Experiment 2 underscore ITap’s ergonomic benefits, particularly evident during prolonged interactions such as scrolling and swiping tasks. ITap significantly reduced physical demands compared to the traditional pinch method, suggesting greater sustainability and practicality for extended usage scenarios. Allowing users to comfortably rest their hands on a tabletop significantly minimized physical exertion and fatigue, aligning with studies emphasizing the ergonomic importance of stable arm support in prolonged VR interactions [16].

Moreover, ITap’s intuitive approach closely aligns with natural human motor control, improving accuracy and reducing cognitive load during scrolling and swiping interactions. The tactile feedback provided by tabletop-based interactions further enhanced usability, contributing to higher precision and reduced error rates, as observed in prior studies on passive haptic feedback in virtual environments [14,37]. These ergonomic and performance benefits clearly highlight ITap’s potential as a preferred interaction method for complex, long-duration XR tasks, supporting existing research advocating for the integration of multimodal inputs to balance cognitive load and accuracy [4,21]. Regarding our second research question, the results strongly support ITap’s advantages. Participants consistently reported greater comfort and reduced cognitive effort using ITap, validating its ergonomic and usability improvements compared to conventional pinch gestures extensively documented in earlier studies [13,20].

Nevertheless, additional experimental work on swiping tasks should be conducted to further enhance performance accuracy. Future research should explore various swiping directions, intensities, and repeated task conditions to comprehensively assess and refine ITap’s usability. Detailed practical experiments will help optimize gesture recognition sensitivity, thus enhancing the user experience across diverse scenarios. Finally, the interpretation of the findings requires refinement to enhance research quality. Future analyses should more explicitly correlate specific ergonomic and cognitive benefits of ITap with quantitative performance metrics, providing clearer insights into the precise mechanisms by which ITap improves user interactions in XR environments.

### 6.3. General Implications and Limitations

Overall, ITap demonstrated clear advantages over the gaze + pinch method across both experiments. These results emphasize the importance of designing XR interaction methods that balance precision, efficiency, and ergonomic considerations, as previously advocated by multimodal interaction research [2,26,34]. Recent studies have further highlighted the growing significance of intuitive gesture-based interactions, emphasizing their advantages in usability and accessibility compared to traditional controller-based methods [38]. Based on the results of this study, ITap is particularly recommended for interactions involving 2D display navigation in VR environments. Its strengths in accuracy and comfort make it especially suitable for tasks such as interacting with home screens, web browsers, application selections, and other 2D panels commonly used in VR scenarios [39]. Future research could explore hybrid interaction approaches dynamically integrating tabletop and mid-air gestures to optimize user comfort and task performance, building upon established frameworks in the literature [20,21].

However, this study has several limitations. Firstly, comparisons were limited to the gaze + pinch method. Future studies should include additional interaction methods, such as gesture-based inputs or controller-based selections, for broader evaluations, as discussed extensively in the literature [19,23,25]. Secondly, variability in gaze-tracking accuracy among individuals and devices was not fully explored. Future research should investigate diverse tracking conditions and user populations to validate ITap’s effectiveness under varied conditions [35,40]. Moreover, additional experimental work on swiping tasks should be conducted to further enhance performance accuracy. Future research should explore various swiping directions, intensities, and repeated task conditions to comprehensively assess and refine ITap’s usability. Detailed practical experiments will help optimize gesture recognition sensitivity and improve user experience across diverse application scenarios.

Additionally, achieving higher analytical accuracy is crucial for real-world applications of ITap. To ensure robust performance, future research should consider advanced analytical methods, including adaptive calibration procedures that dynamically adjust to individual user characteristics and environmental conditions. Incorporating machine learning algorithms could also enhance gesture recognition precision and responsiveness, further improving real-world usability. Such adaptive approaches could further optimize interaction experiences tailored to individual user needs, thereby improving overall user satisfaction and inclusivity.

## 7. Conclusions

This study evaluated the effectiveness of ITap, a novel interaction method integrating gaze input with tabletop-based finger interactions, comparing it directly with the traditional gaze + pinch method across various scenarios. The research specifically addressed two primary research questions: (1) Does ITap’s manual mode offer superior target selection performance compared to the traditional gaze-based gesture (gaze + pinch)? (2) Does ITap provide improved usability and reduced cognitive load compared to the Pinch gesture?

Based on empirical results, both research questions were affirmatively addressed. ITap consistently demonstrated superior performance in target selection tasks, particularly at medium to long distances and in densely arranged scenarios, by significantly reducing selection times and error rates compared to gaze + pinch. This enhanced performance was primarily due to ITap’s manual mode, which allowed users to efficiently correct gaze inaccuracies through intuitive finger movements, ensuring precise and reliable interactions. Moreover, ITap provided marked ergonomic and usability improvements during prolonged interaction tasks such as scrolling and swiping. Participants experienced less fatigue and reported higher levels of comfort and naturalness, indicating that ITap effectively reduces physical and cognitive demands commonly associated with mid-air interaction methods.

The findings emphasize that ITap represents a significant advancement in interaction techniques, particularly suited to realistic and high-demand VR applications involving extensive 2D interface navigation. In practical contexts, ITap can be effectively applied to common VR tasks including home interface navigation, web browsing, app selection, and interactions with various 2D panels. Unlike conventional gaze interaction methods that frequently induce physical strain (“gorilla arm syndrome”) due to prolonged mid-air arm elevation, ITap’s tabletop interaction provides stable hand support, enhancing both usability and comfort. Such ergonomic advantages facilitate extended use, thereby greatly improving user satisfaction and productivity.

However, the current research identified limitations in ITap’s applicability within three-dimensional (3D) interaction contexts, as selecting and manipulating objects in 3D environments inherently introduces additional complexity. Future research should address these limitations by developing advanced techniques specifically tailored to enhance ITap’s capabilities in three-dimensional scenarios. Possible approaches include dynamic repositioning or resizing of the virtual touchpad to accommodate 3D spatial interactions, and the integration of two-handed gesture recognition technologies to support more sophisticated, intuitive, and complex interaction patterns.

Additionally, to further refine ITap’s applicability and precision, future studies should incorporate adaptive calibration methods leveraging machine learning algorithms. These methods could dynamically account for individual variations in gaze and hand-tracking accuracy, significantly enhancing real-world application performance. Moreover, exploring hybrid interaction models that seamlessly blend tabletop-based and mid-air interactions could provide flexible and optimized user experiences across diverse VR and AR scenarios.

## Data Availability

The dataset supporting the results of this study is openly available on Figshare at: https://figshare.com/articles/dataset/ITap_Index_Finger_Tap_Interaction_by_Gaze_and_Tabletop_Integration/28435211?file=52431923 (accessed on 4 March 2025).
